# The Treatment of Nævi by Electricity

**Published:** 1902-05-24

**Authors:** 


### Hospital Clinics and Medical Progress.

### THE TREATMENT OF NÆVI BY ELECTRICITY.

In discussing the treatment of nævi by electricity Dr. Lewis Jones[1] points out that we may either employ the electricity directly, passing the current into the tissues and so destroying the nævus by electrolysis, or indirectly by using it to produce heat in a galvano-cautery. Many cases can be treated very nicely with the galvano-cautery. The heat to aim at is a dull red. If too hot the cautery will cut the tissues and they will bleed, while if not hot enough they will stick to it. Again, one must not be tempted merely to rub the cautery over the surface of the nævus. By so doing an intense constriction of the blood-vessels will be produced which makes the nævus seem as if it were destroyed, but it will be found that it is not so when the scar comes away. It is necessary to push the cautery through into the nævus and to make sure that it goes as deeply as the nævoid tissues extend. When electrolysis is used the nævus is destroyed, not by heat, but by chemical action, the caustic chemical substances which are liberated and accumulate round the needles devitalising the tissues. The amount of these caustic substances liberated is proportional to the amount of the current which passes through the nævus and to the length of time during which it is allowed to pass. At the positive pole there are hydrochloric, phosphoric, and sulphuric acids; while at the negative pole there are potash and soda, and so forth. It is these chemicals which do the work and not the flow of current from one pole to the other. One may introduce both poles into the nævus, or one may use only one, the circuit being completed through the patient by means of a pad wetted with water applied to some other part. One disadvantage of using a pad is that there may be a certain amount of shock in some cases from the passage of the current from the pad through the body—through the neck, for example, when the nævus is on the cheek or nose—and by placing both poles in the nævus one avoids this risk of a shock. But it may not always be convenient to introduce both poles into the nævus. If it is small one does not want a strong current, and one does want to do fine work; in this case, therefore, one may use a single needle and a pad, but with larger nævi it is best to introduce both poles. The difference in action between the positive and negative poles, although very appreciable, is not important; both poles destroy—the positive by the acids, the negative by the alkalies which are liberated. The positive pole which liberates acids coagulates the tissues around it, and consequently adheres to them, one result of which is that in withdrawing the needle bleeding may occur. Very seldom, however, is there any bleeding from the negative pole, and on this account when using only one needle for fine work it should be the negative one. The battery which Dr. Jones showed contained 30 small Leclanche cells, but he said that for doing electrolysis one seldom required more than 12 or 15 of them. As to the magnitude of the current used, if one watches the galvanometer during the electrolysis one finds that the operation requires from 20 to 30 milliampères. But it is the density of the current in the neighbourhood of the needles, or the strength per unit of needle surface, which determines the amount of destructive action, so that the larger the surface of the needles which is buried the larger is the current required. A simple rule to lay down is that the current should be about 30 milliampères per inch of positive or negative pole which is buried. The aim of the treatment is not to kill the whole tissues of the nævus—which would cause it to slough, producing an ugly scar—but to produce plastic inflammation, keeping short of complete destruction. By so doing one produces a firm inflamed area and obliterates the nævus at the expense of very little scarring. To know how far one may go there is nothing like practice. If one watches the process one sees a little greyish zone forming round the points of entry of the needles. This gets bigger the longer the current is continued, and Dr. Jones says, "If you change the position of the needles when this zone is about one-eighth of an inch across you will get destruction with very little scarring at that needle hole, but if you wait until it is a quarter of an inch across you will have a sloughing there, and there will be a scar left afterwards. So if the nævus is large enough to require it, change the position of your needles during the electrolysis, and introduce them afresh until you have completely covered the ground." It is by its edge that a nævus grows, so one must insert the needles round the margin, a little outside the edge rather than inside it. A large nævus is much more difficult to deal with than a small one, and when one is once convinced that a nævus is growing the sooner it is removed the better.

[1] Clinical Journal, April 20.

